# Supplementary material for: The Impact of Medical Nutrition Intervention on the Management of Hyperphosphatemia in Hemodialysis Patients with Stage 5 Chronic Kidney Disease: A Case Series
Source: Int J Environ Res Public Health. 2023 Mar 13;20(6):5049. doi: 10.3390/ijerph20065049 (PMC10049720; doi:10.3390/ijerph20065049)
Supplement: Supplementary file 1 [file ijerph-20-05049-s001.zip › Supplementary Material Tables S1-S6 and Figure S1.docx]

**Table S1.** Friedman test for calcemia values

| **Test Statistics^a^** | |
| --- | --- |
| N | 18 |
| Chi-Square | 5.333 |
| df | 2 |
| Asymp. Sig. | 0.069 |
| a. Friedman Test | |

**Table S2**. Wilcoxon test for calcemia values

| **Ranks** | | | | |
| --- | --- | --- | --- | --- |
| Wilcoxon Signed Ranks Test | | N | Mean Rank | Sum of Ranks |
| Calcemia from measurement 3 (mg/dL) – Calcemia from measurement 1 (mg/dL) | Negative Ranks | 8^a^ | 12.31 | 98.50 |
|  | Positive Ranks | 10^b^ | 7.25 | 72.50 |
|  | Ties | 0^c^ |  |  |
|  | Total | 18 |  |  |
| a. Calcemia from measurement 3 (mg/dL) < Calcemia from measurement 1 (mg/dL) | | | | |
| b. Calcemia from measurement 3 (mg/dL) > Calcemia from measurement 1 (mg/dL) | | | | |
| c. Calcemia from measurement 3 (mg/dL) = Calcemia from measurement 1 (mg/dL) | | | | |
| \| **Test Statistics^a^ Results** \| \| \| --- \| --- \| \|  \| Calcemia from measurement 3 (mg/dL) – Calcemia from measurement 1 (mg/dL) \| \| Z \| -.566^b^ \| \| Asymp. Sig. (2-tailed) \| 0.571 \| \| ^a^ Wilcoxon Signed Ranks Test \| \| \| ^b^ Based on positive ranks. \| \| | | | | |

| 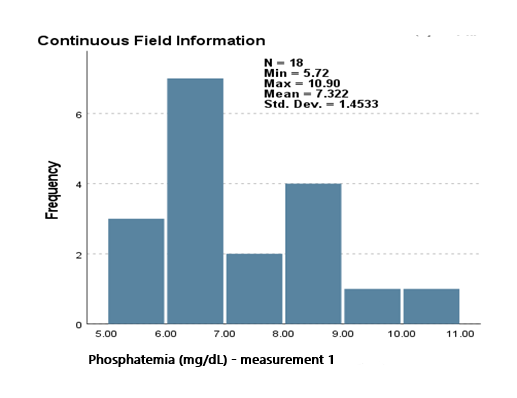 |
| --- |
| (a) |
| 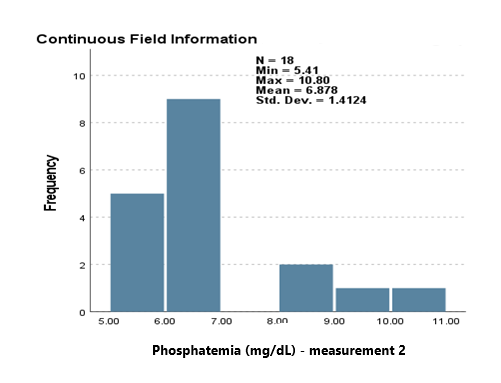 |
| (b) |
| 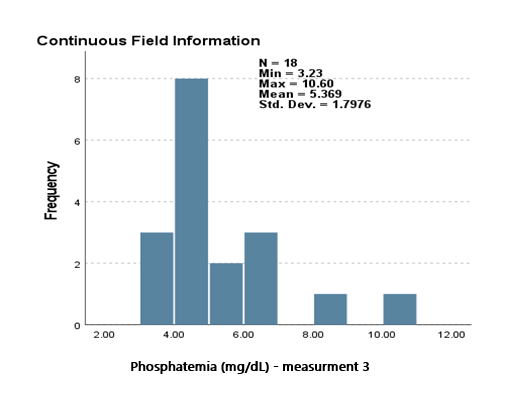 |
| (c) |

**Figure S1.** The evolution of phosphatemia levels on all three measurements (a) first measurement, (b) second measurement, and (c) third measurement, where Frecvency = number of individuals

**Table S3.** Friedman test for phosphatemia values

| **Test Statistics^a^** | |
| --- | --- |
| N | 18 |
| Chi-Square | 16.778 |
| df | 2 |
| Asymp. Sig. | 0.000227 |
| a. Friedman Test | |

**Table S4**. Wilcoxon test for phosphatemia values

| **Wilcoxon Signed Ranks Test** | | N | Mean Rank | Sum of Ranks |
| --- | --- | --- | --- | --- |
| Fosfor seric Luna3 (mg/dl) - Fosfor seric Luna 1 (mg/dl) | Negative Ranks | 16^a^ | 10.31 | 165.00 |
|  | Positive Ranks | 2^b^ | 3.00 | 6.00 |
|  | Ties | 0^c^ |  |  |
|  | Total | 18 |  |  |
| a. Phosphatemia measurement 3 (mg/dL) < Phosphatemia measurement 1 (mg/dL) | | | | |
| b. Phosphatemia measurement 3 (mg/dL) > Phosphatemia measurement 1 (mg/dL) | | | | |
| c. Phosphatemia measurement 3 (mg/dL) = Phosphatemia measurement 1 (mg/dL) | | | | |
| \| **Test Statistics^a^** \| \| \| --- \| --- \| \|  \| Fosfor seric Luna3 (mg/dl) - Fosfor seric Luna 1 (mg/dL) \| \| Z \| -3.462^b^ \| \| Asymp. Sig. (2-tailed) \| <.001 \| \| a. Wilcoxon Signed Ranks Test \| \| \| b. Based on positive ranks. \| \| | | | | |

**Table S5.** Friedman Test for phosphate chelator drugs’ dose evolution

| **Test Statistics^a^** | |
| --- | --- |
| N | 18 |
| Chi-Square | 26.000 |
| df | 2 |
| Asymp. Sig. | 0.000 |
| a. Friedman Test | |

**Table S6.** Wilcoxon test for phosphate chelator drugs’ dose evolution

| **Ranks** | | | | |
| --- | --- | --- | --- | --- |
| Wilcoxon Signed Ranks Test | | N | Mean Rank | Sum of Ranks |
| Phosphate chelator drug measurement 3 (type, dose) - Phosphate chelator drug measurement 1(type, dose) | Negative Ranks | 13^a^ | 7.00 | 91.00 |
|  | Positive Ranks | 0^b^ | 0.00 | 0.00 |
|  | Ties | 5^c^ |  |  |
|  | Total | 18 |  |  |
| a. Phosphate chelator drug measurement 3 (type, dose) < Phosphate chelator drug measurement 1(type, dose) | | | | |
| b. Phosphate chelator drug measurement 3 (type, dose) > Phosphate chelator drug measurement 1 (type, dose) | | | | |
| c. Phosphate chelator drug measurement 3 (type, dose) = Phosphate chelator drug measurement 1 (type, dose) | | | | |
| \| **Test Statistics^a^** \| \| \| --- \| --- \| \|  \| Phosphate chelator drug measurement 3 (type, dose) - Phosphate chelator drug measurement 1 (type, dose) \| \| Z \| -3.228^b^ \| \| Asymp. Sig. (2-tailed) \| 0.001 \| \| a. Wilcoxon Signed Ranks Test \| \| \| b. Based on positive ranks. \| \| | | | | |
